# Pyroptosis in renal inflammation and fibrosis: current knowledge and clinical significance

**DOI:** 10.1038/s41419-023-06005-6

**Published:** 2023-07-27

**Authors:** Ya Liu, Haibo Lei, Wenyou Zhang, Qichang Xing, Renzhu Liu, Shiwei Wu, Zheng Liu, Qingzi Yan, Wencan Li, Xiang Liu, Yixiang Hu

**Affiliations:** 1 Molecular Pharmacology Laboratory, Department of Clinical Pharmacy, Xiangtan Center Hospital, Xiangtan, 411100 China; 2 Honghao Zhou Research Institute, Xiangtan Center Hospital, Xiangtan, 411100 China; 3 Department of Pharmacy, Sir Run-Run Shaw Hospital, Zhejiang University School of Medicine, Hangzhou, 310006 China

**Keywords:** Inflammasome, Chronic kidney disease

## Abstract

Pyroptosis is a novel inflammatory form of regulated cell death (RCD), characterized by cell swelling, membrane rupture, and pro-inflammatory effects. It is recognized as a potent inflammatory response required for maintaining organismal homeostasis. However, excessive and persistent pyroptosis contributes to severe inflammatory responses and accelerates the progression of numerous inflammation-related disorders. In pyroptosis, activated inflammasomes cleave gasdermins (GSDMs) and generate membrane holes, releasing interleukin (IL)-1β/18, ultimately causing pyroptotic cell death. Mechanistically, pyroptosis is categorized into caspase-1-mediated classical pyroptotic pathway and caspase-4/5/11-mediated non-classical pyroptotic pathway. Renal fibrosis is a kidney disease characterized by the loss of structural and functional units, the proliferation of fibroblasts and myofibroblasts, and extracellular matrix (ECM) accumulation, which leads to interstitial fibrosis of the kidney tubules. Histologically, renal fibrosis is the terminal stage of chronic inflammatory kidney disease. Although there is a multitude of newly discovered information regarding pyroptosis, the regulatory roles of pyroptosis involved in renal fibrosis still need to be fully comprehended, and how to improve clinical outcomes remains obscure. Hence, this review systematically summarizes the novel findings regarding the role of pyroptosis in the pathogenesis of renal fibrosis and discusses potential biomarkers and drugs for anti-fibrotic therapeutic strategies.

## Facts

- Half of all adults over 70 and 10% of the world’s population suffer from chronic kidney disease and renal fibrosis.
- Excessive and persistent inflammation has been identified as a crucial mechanism in the formation of renal fibrosis.
- As a novel inflammatory form of regulated cell death, pyroptosis has emerged as a crucial inducer of the inflammatory process and plays an essential role in the development of renal inflammation and fibrosis.
- An in-depth understanding of the mechanisms that regulate pyroptosis will allow for the discovery of novel targeted inhibitors for renal fibrosis.

## Open questions

- Mechanisms and significance of pyroptosis in renal inflammation and fibrosis.
- Reliable and sensitive pyroptosis biomarkers for renal fibrosis.
- What if targeting GSDMD and GSDME could be a valuable strategy for treating renal fibrosis?
- If GSDMD, NLRP3, IL-1β, Caspase-1, and P2X7R inhibitors could prevent renal inflammation and fibrosis in humans?

## Introduction

Several pathogenic mechanisms destroy kidney structural and functional units and irreversibly reduce kidney function in chronic kidney disease (CKD) [1]. Histologically, regardless of the initial damage, renal fibrosis is the terminal stage of CKD [2]. Aside from kidney transplantation and dialysis, renal fibrosis has no effective treatment. Consequently, it is essential to comprehend the mechanisms of renal fibrosis in order to discover novel targets and drugs that can prevent this pathological process.

Inflammation is regarded as a host defensive mechanism against pathogens, which can generate inflammatory cytokines to activate innate immunity in response to relevant stimuli. In addition to bacterial and viral infections, lipid metabolism, high glucose levels, and ischemia-reperfusion injury also trigger the onset of renal inflammation [3]. Excessive and persistent inflammation has been identified as a crucial mechanism in the formation of renal fibrosis [4]. The release of inflammatory cytokines and pro-fibrotic factors during CKD progression promotes the development of renal fibrosis [5]. Pyroptosis, a novel inflammatory form of regulated cell death (RCD), is distinct from necroptosis, apoptosis, autophagy, ferroptosis, and cuproptosis in morphological characteristics. When initiated, pyroptosis eliminates fungal, bacterial, and viral pathogens by a powerful inflammatory response essential for organismal homeostasis [6]. Numerous research has demonstrated that pyroptosis is driven by caspase-1/4/5/11 in response to cellular injury [7]. This process correlates with the inflammasome activation, a molecular platform that results in the release of cleaved-caspase-1 and the production of interleukin (IL)-1β/18 in response to intracellular and extracellular stimuli [8]. The gasdermins (GSDMs) also possess an essential function in the progression of pyroptosis. After being cleaved by upstream proteases, the GSDMs trigger pyroptotic cell death by their pore-forming activities. As a substrate of caspase-1/4/5/11, GSDMD is the principal pyroptotic executioner [9]. Active caspase-1 cleaves the Asp275 domain of GSDMD to produce an N-terminal GSDMD fragment (GSDMD-NT), which mediates the formation of pyroptotic pores in cell membranes and causes a pro-inflammatory effect [10, 11]. However, inflammasome and GSDMD activation do not necessarily result in considerable cell lysis. According to a recent study, pyroptosis traps living bacteria but does not trigger cell lysis until phagocytes consume them [12].

Emerging evidence suggests that pyroptosis plays a significant role in the development of renal inflammation and fibrosis [13]. However, the detailed mechanism of pyroptosis in this disease has yet to be entirely understood. The purpose of this review was to summarize the roles and plausible mechanisms of pyroptosis in renal inflammation and fibrosis. Eventually, based on the existing advancements, prospective therapeutic targets of pyroptosis and future directions will also be inferred.

## Chronic inflammation and renal fibrosis

Renal fibrosis is a prominent pathological characteristic of end-stage chronic kidney disorders. Early phases of renal fibrosis are characterized by multiple inflammatory cytokines and growth factors involving innate and adaptive immune responses [14]. This mechanism includes three stages that overlap: 1) the wound healing phase, 2) the inflammatory phase, and 3) the proliferative phase or remodeling/maturation phase [15]. This inflammatory process is transmitted by epithelial and endothelial cells, triggering the recruitment of inflammatory cells, including macrophages, lymphocytes, mast cells, eosinophils, and basophils. Notably, both M1 and M2 macrophages are required for the early inflammatory phase of the wound healing response [15]. Initially, M1 macrophages release pro-inflammatory cytokines such as tumor necrosis factor-alpha (TNF-α), IL-1β/18, and chemokine CCL2 to trigger the early inflammatory phase [16]. On the other hand, transforming growth factor-beta (TGF-β) produced by kidney-infiltrating M2 macrophages exacerbates the progression of renal fibrosis in the remodeling phase [16]. Of note, it has been revealed that TGF-β is a potent chemoattractant that recruits M2 macrophages and mediates NLRP3 activation to cause tubule-interstitial fibrosis [16]. Accordingly, macrophage depletion is advantageous and ameliorates renal fibrosis following diverse traumas.

The transition of CKD to end-stage renal disease is closely associated with the increased deposition of collagens and other extracellular matrices (ECM) proteins, which occurs concurrently with the loss of typical kidney architecture [17]. It is well documented that the fibrotic lesions of glomerular tuft are induced mostly by laminin (LN) and type I, III, and IV collagen proteins, reducing the glomerular blood flow and filtration capacity [18]. Quiescent fibroblasts situated in the interstitial space play an essential function in preserving the kidneys’ structural integrity by generating a baseline amount of ECM [18]. However, in response to various pro-inflammatory cytokines and pro-fibrotic factors, fibroblasts convert into myofibroblasts during chronic inflammatory stimulation in the kidneys [18]. In general, myofibroblasts display atypical pro-inflammatory phenotype and secrete multiple chemokines, including α-smooth muscle actin (SMA), matrix metalloproteinases, and tissue inhibitor of metalloproteinase, therefore have pathogenic functions that result in renal fibrosis [19]. Collectively, addressing inflammation and fibrosis is a top priority for preventing kidney injury.

## Overview of pyroptosis and comparison with other regulated cell death modalities

**Fig. 1:**
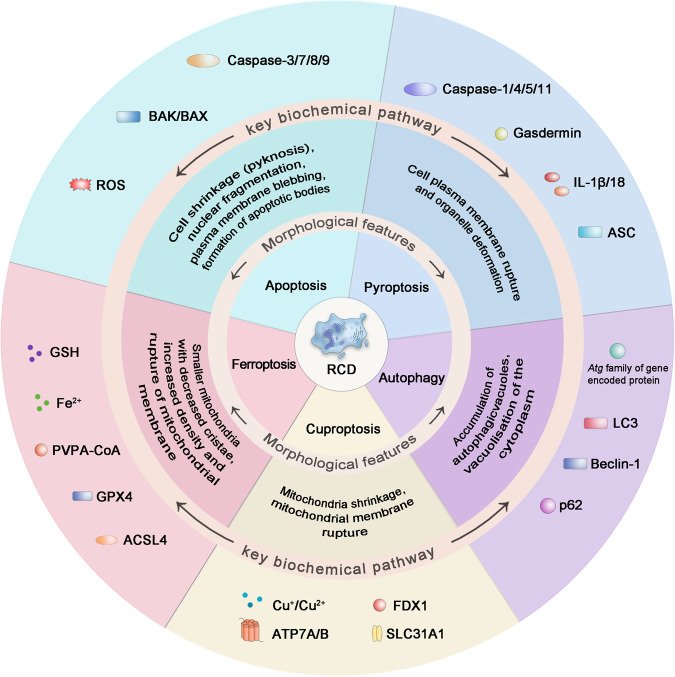
Morphological characteristics and key biochemical pathway components of regulated cell death pathways. GSDMD gasdermin D, ASC adaptor molecule apoptosis associated speck-like protein, IL-1β/18 interleukin-1β/18, LC3 microtubule-associated protein light chain 3, FDX1 mitochondrial enzyme ferredoxin 1, SLC31A1 solute carrier family 31 member 1, ATP7A/B copper-transporting ATPase 1/2, GSH glutathione, GPX4 glutathione peroxidase 4, ACSL4 acyl-CoA synthetase long-chain family member 4, LIAS lipoyl synthase, ROS reactive oxygen species.

The cell death process can be classified into RCD and accidental cell death based on physical appearance, biological function, and regulatory mechanisms. Apoptosis, autophagy, ferroptosis, cuproptosis, and pyroptosis were among the RCD mechanisms defined by the Nomenclature of Cell Death Committee [20]. These RCD subroutines have distinct traits while exhibiting several comparable features and substantial overlap and crosstalk (Fig. 1). Apoptosis is a form of RCD that occurs through the regulation of intracellular genes and is typically dependent on the activity of non-inflammatory proteases such as caspase-3/7/9 in the caspase family [21]. As a highly conserved metabolic process, autophagy regulates the catabolism of biomolecules by transferring damaged organelles and abnormal proteins to lysosomes and degrading them to produce new metabolic substrates [22]. Ferroptosis is a newly discovered iron-dependent RCD, which is mainly related to iron-dependent lipid peroxidation and glutathione exhaustion, leading to ROS generation and cell death [23]. The latest research indicates that copper induces cuproptosis by binding to lipid-acylated components of the tricarboxylic acid cycle, causing protein aggregation, loss of iron–sulfur cluster proteins, and ultimately proteotoxic stress [24]. Disulfidptosis, a novel RCD form discovered in 2023, induces cell death by accumulating cystine and disulfide stress; however, its exact mechanism for regulating cell death requires further study [25]. In contrast to other forms of RCD, pyroptosis is a well-known pro-inflammatory RCD mode associated with the innate immune system [26].

**Fig. 2:**
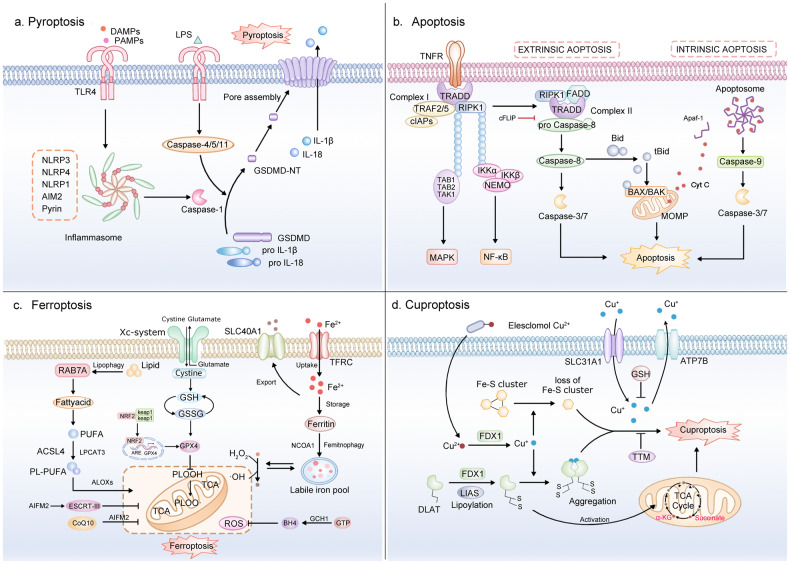
Pathways controlling pyroptosis, apoptosis, ferroptosis, and cuproptosis. **a** Pyroptosis can be triggered in response to PAMPs or DAMPs, which activate inflammasomes such as NLRP1, NLRP3, NLRP4, AIM2, and Pyrin and recruit the adaptor ASC, resulting in caspase-1 activation. LPS activates Caspase-4/5/11 in the cytoplasm. Ultimately, active caspase-1/4/5/11 cleaves GSDMD to generate GSDMD-NT, resulting in pore formation, plasma membrane rupture, cell lysis, and IL-1β/18 release. **b** Apoptosis can occur via an intrinsic or extrinsic pathway. The intrinsic pathway is activated by mitochondrial damage. Cytochrome c induces apoptotic protease activating factor-1 (Apaf-1) and a caspase-9 precursor combine to produce an apoptosome that activates caspase-9. In turn, caspase 9 activates caspase-3/7, resulting in apoptosis. In the extrinsic pathway, the oligomerization of cell surface death receptors leads to caspase-8 activation, which directly cleaves pro-caspase-3 to mediate apoptosis. Additionally, caspase-8 can cleave Bid to produce tBid, which migrates to the mitochondria and forms Bax/Bak openings on its surface, releasing cytochrome c and initiating apoptosis. **c** Ferroptosis is an iron-dependent RCD, mainly related to iron-dependent lipid peroxidation and glutathione (GSH) exhaustion, leading to ROS generation and cell death. The ferroportin (SLC40A1), transferrin-transferrin receptor complex (TF-TFRC), and ferritinophagy contribute to iron accumulation and cause ferroptosis. On the other hand, in conjunction with RAB7A-dependent lipophagy, the ACSL4/LPCAT3/ALOXs pathway promotes ferroptosis by activating lipid peroxidation to generate PLOOH. GPX4 and other free radical scavengers, such as ferrostatin-1 inhibit lipid peroxidation (lipid-ROS). In the absence of GPX4, lipid peroxides accumulate, resulting in the onset of ferroptosis. **d** Cuproptosis can be induced by accumulation of intracellular free copper ions. In particular, elesclomol acts as a copper ionophore that facilitates copper transport into cells. Intracellular copper levels are also regulated by copper importers (SLC31A1) and copper exporters (ATP7B). FDX1, a reductase, is responsible for reducing Cu^2+^ to Cu^+^ which helps in the lipoylation of mitochondrial TCA cycle enzymes, particularly DLAT. This process leads to Fe-S cluster protein instability, causing proteotoxic stress and ultimately cell death.

Pyroptosis was initially noticed in 1992; however, it was first misidentified as morphological alterations of apoptosis [27]. A decade later, in 2001, after observing salmonella-mediated macrophage death, Brennan termed this new form of cell death pyroptosis [28]. Pyroptosis has been observed in various cell types, including monocytes/macrophages, hepatocytes, endothelial cells, and renal tubular epithelial cells [29–31]. The classical morphology of pyroptosis includes cell swelling and cell membrane rupture mediated by GSDMs, which ultimately leads to the secretion of intracellular contents such as inflammatory factors and lactate dehydrogenase. These characteristics distinguish pyroptosis from other RCD forms (Fig. 2). Chromatin coagulation occurs in both pyrosis and apoptosis; however, the nucleus remains intact in pyroptosis, and karyorrhexis does not occur [32]. Both apoptosis and pyroptosis are initiated by caspases. Biochemically, caspase-3/7/9 are involved in apoptosis, while pyroptosis is induced by pro-inflammatory caspases-1/4/5/11 [33–35]. Compared to pyroptosis, ferroptosis exhibits a distinct feature of increased density and rupture of the mitochondrial membrane and does not necessitate caspase activation [23]. From a cellular perspective, pyroptosis and cuproptosis also have distinct characteristics. Cuproptosis can be triggered by intracellular free copper ions and is characterized by a reduction in the mitochondrial crest and mitochondrial membrane lysis [36]. In contrast, pyroptosis is distinguished by the formation of inflammasomes, GSDMs-dependent cell membrane rupture, and release of IL-1β/18.

## Activation of pyroptosis

The host can detect intracellular and extracellular threats that are created by invading microbes or tissue damage. The pattern-recognition receptors (PRRs) encoding the germline activated by the innate immune system recognize invariant microbial patterns [37]. PRRs are typically found in monocytes, dendritic cells, and epithelial and neutrophil cells, where they serve as infection markers to detect damage-associated molecular patterns (DAMP) and pathogen-associated molecular patterns (PAMPs) [38]. Based on their location, PRRs can be categorized into C-type lectin-like receptors (CLRs) and Toll-like receptors (TLRs), nucleotide-binding oligomerization domain-like receptors (NLRs), retinoic acid-inducible gene I-like receptors, and AIM2-like receptors (ALRs) [39]. Among them, the TLR and CLR recognize PAMPs, while the NLR recognizes both PAMPs and DAMPs. The NLR is involved in the identification of host cell danger signals, whereas TLRs initiate an inflammatory reaction that stimulates cells and produces inflammatory cytokines [40].

## Inflammasomes and pyroptosis

**Fig. 3:**
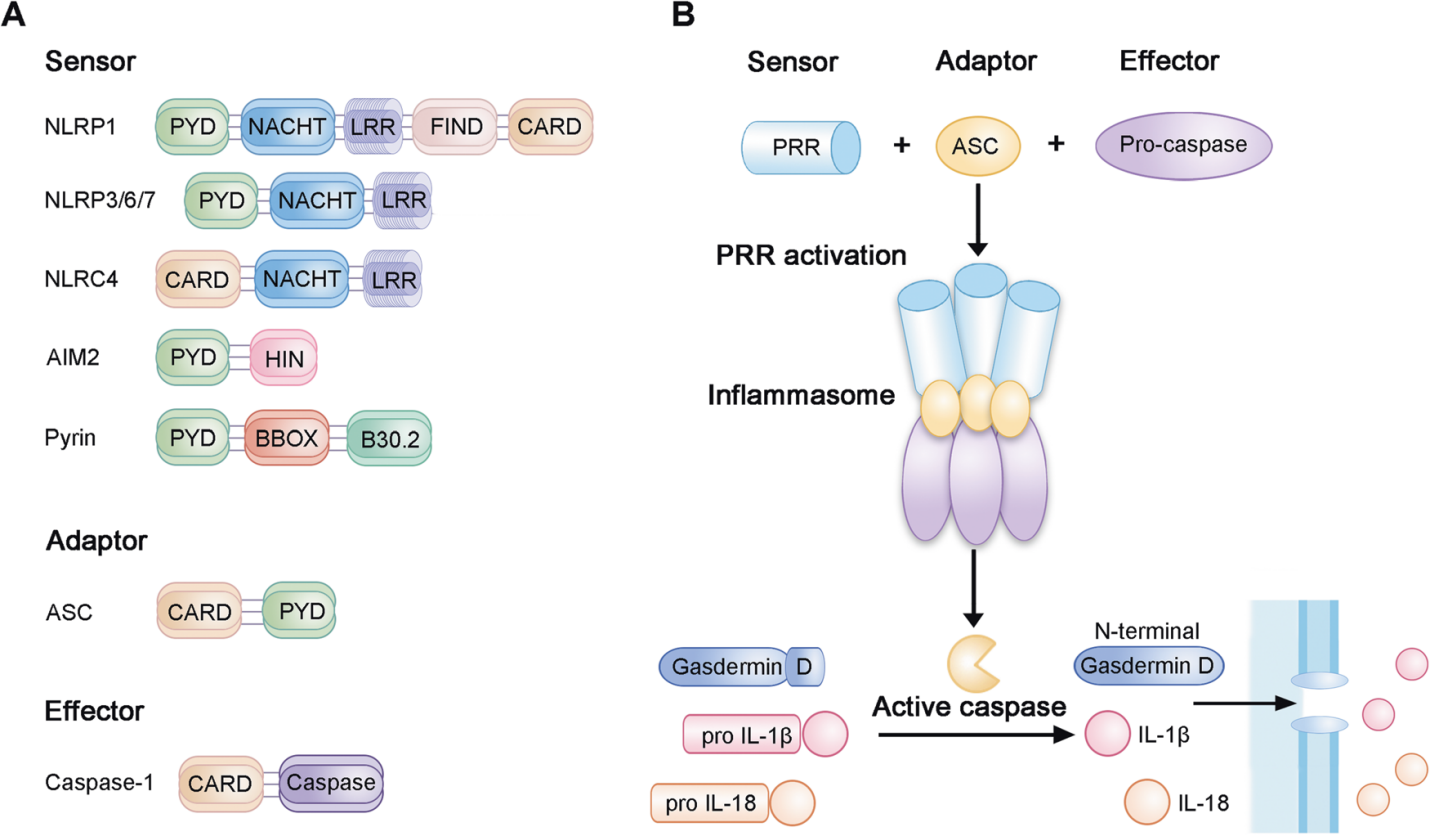
Inflammasome assembly and molecules that can trigger pyroptosis. **a** The NOD-like receptor (NLR) family has common PYD and NACHT domains, C-terminal leucine-rich repeat (LRR), and the N-terminal effector domains. The AIM2 receptor possesses a PYD and HIN200 domain, while pyrin possesses a PYD, two B boxes, and a B30.2 domain. Adaptor possesses a PYD for combining with the sensor and a CARD for combining with the effector, whereas the effector consists of both a CARD and pro-caspase-1. **b** As the molecular switch of pyroptosis, the inflammasome complex consists of a sensor, adaptor, and effector protein such as pro-caspase-1. The active inflammasome cleaves the effector. After being cleaved by active caspase, the N-terminal GSDMD fragment triggers pyroptotic cell death by their pore-forming activities, allowing the secretion of IL-1β/18.

Inflammasomes play a vital role in initiating inflammation and pyroptosis. After activation, inflammasomes form a big complex composed of an oligomerizing sensor protein, an adaptor protein, and an effector protein [41]. Sensor proteins mainly comprise NLRs, HIN200 protein absent in melanoma 2 (AIM2), and pyrin, which share analogous structural domains [42]. Specifically, pyroptosis-related NLRs include NLRP1/3/6/7 and NLRC4 (Fig. 3).

In humans, functional NLRP1 is mainly expressed in epithelial barrier tissues, including bronchial epithelial cells and keratinocytes. In vitro analysis revealed that NLRP1 is a component of a high-molecular-weight complex that serves as a platform for activating caspase-1 [43]. It was demonstrated that recombinant NLRP1 oligomerizes in response to component muramyl dipeptide, thereby activating caspase-1 and pyroptosis [43].

The NLRP3 inflammasome was identified as the molecule most closely associated with the initiation of pyroptosis [44]. It comprises an adaptor molecule called apoptosis-associated speck-like protein (ASC), caspase-1, and a basic NLR scaffold containing a central NACHT domain. The NLRP3 inflammasome responds to a wide range of stimuli, which causes its activation and plays a vital role in different signaling pathways. Under physiological conditions, the pro-inflammatory response elicited by the NLRP3 inflammasome serves as a protective mechanism for the organism and contributes to homeostasis maintenance [45]. The stimuli involved in NLRP3 activation include PAMPs (e.g., fungal, bacterial, viruses, nucleic acids, and pore-forming toxins) and DAMPs (e.g., extracellular ATP, amyloid, and uric acid crystals) [46]. Two distinct steps are required for NLRP3 inflammasome activation. Firstly, priming is mediated by microbial ligands recognized by TLRs, which activate the TLR4/Myd88/NF-κB pathway and stimulate the synthesis of pro-IL-1β/18 and NLRP3 proteins [46]. After that, secondary stimuli such as ATP trigger the assembly of the inflammasome complex and cleavage of the pro-IL-1β/18 [47]. In addition to mediating the inflammatory response, the NLRP3 inflammasome is also involved in pyroptosis, mitochondrial regulation, and the transformation and proliferation of myofibroblasts during renal fibrosis [48, 49]. With more profound research, the activation of NLRP3 inflammasome has been implicated in the progression of renal fibrosis, all of which interact with complicated metabolic alterations in the kidneys [50].

NLRP6 and NLRP7 inflammasomes are members of the pyroptosis-related NLRs with structural analogies [51, 52]. The NLRP6 inflammasome is primarily expressed in the gastrointestinal tract [51]. It was discovered that the NLRP6 inflammasome could promote the pathogenesis and progression of post-inflammatory irritable bowel syndrome (PI-IBS) [51]. Additionally, the NLRP6 inflammasome has a protective effect against renal injury and could inhibit the progression of renal fibrosis [53]. NLRP7 was discovered to be associated with the disease in 1999 [52]. According to recent research, the NLRP7 inflammasome can promote colorectal cancer progression and mediate tumor-associated macrophage polarization [54].

NLRC4 is essential for recognizing Gram-negative bacteria in the cytoplasm. Although it is evident that NLRC4 is expressed and active in intestinal epithelial cells, the role of NLRC4 in immune cells remains controversial [55]. Of note, mitophagy might activate the NLRC4 inflammasome and contribute to kidney damage via the generation of mROS [56]. However, the putative activation processes and function of the NLRC4 inflammasome in renal fibrosis remain unknown.

As an inflammasome component, AIM2 is a cytoplasmic double-stranded DNA sensor in the innate immune cell. Initially, AIM2 was identified as a tumor suppressor for hepatocellular carcinoma, melanoma, and HPV-infected cervical carcinoma [57–59]. Recent research indicates that phagocytosed dsDNA can be detected by AIM2 in the cytoplasm of macrophages, contributing to the release of IL-1β/18 and leading to pyroptosis [60]. However, despite several studies indicating the role of AIM2 in renal inflammation and fibrosis, it is still unknown whether it operates via the pyroptosis pathway.

Pyrin expression is predominantly restricted to immune cells, specifically granulocytes, eosinophils, macrophages, and monocytes [61]. It has been recognized that the pyrin inflammasome mediates inflammation in various inflammatory illnesses associated with the deregulation of the actin polymerization pathway [62]. However, no evidence demonstrates its direct influence on renal inflammation and fibrosis progression.

## Gsdms and pyroptosis

In 2015, the ability of GSDMs to trigger pyroptosis was identified [63]. Lately, significant research has been published to understand its involvement in the control of the inflammatory response and the physiopathology of a number of chronic disorders, including chronic cholecystitis and renal fibrosis [64, 65]. With in-depth research, the understanding of pyroptosis has gradually evolved from the original definition of caspase-1-dependent RCD to GSDMs-dependent RCD. In humans, GSDMs have six members: GSDMs A-E and DFNB59.

GSDMA is associated with autoimmune disorders and malignancies [66]. Recently, it has been discovered that GSDMA gene deficiency impairs mouse immune responses, leading to uncontrolled bacterial transmission and mortality [67]. Additionally, the Streptococcus pyogenes cysteine protease SpeB virulence factor induces keratinocyte pyroptosis by cleaving GSDMA [67]. Nonetheless, further investigation is needed to determine whether other proteases could activate GSDMA.

GSDMB is expressed in several different human tissues, such as the small intestine, colon tissue, stomach, and liver, but it is the only gasdermin not found in rodents [68]. This indicates that GSDMB is not a typical mammalian immune system component but has evolved a specialized function in humans and other mammals. Specifically, GSDMB polymorphisms were related to a higher risk of inflammatory disorders with complicated traits, including PI-IBS, ulcerative colitis, and asthma [69–71]. In contrast to other GSDMs, both full-length and cleaved GSDMB can induce pyroptosis [72]. In inflammatory bowel disease patients, GSDMB levels are increased, and caspase-1 cleaves GSDMB to trigger epithelial cell pyroptosis [68]. Nonetheless, the function of GSDMB-mediated pyroptosis in developing renal inflammation and fibrosis is poorly understood.

GSDMC is mainly expressed in the skin, spleen, vagina, and esophagus [73]. However, no activation mechanisms that activate GSDMC have been identified. Current research revealed that GSDMC is a promising oncogene, promoting colorectal cancer cell proliferation by inhibiting TGFBR2 activation [74]. Additionally, the overexpression of GSDMC triggered by programmed death-ligand 1 transforms apoptosis into pyroptosis in tumor cells [75]. Meanwhile, the metabolite α-KG was discovered to initiate GSDMC-dependent pyroptosis via activating caspase-8 [76].

GSDMD is the first GSDMs discovered to be involved in pyroptosis [63]. Regarding function and mechanism, GSDMD is the primary executor of pyroptosis [63]. GSDMD consists of a C-terminal domain (GSDMD-CT) and a GSDMD-NT, which are connected by a 43-amino acid bridge [63]. GSDMD-NT can trigger pyroptosis via pore-forming activities, whereas GSDMD-CT can protect cells against pyroptosis under non-stimulating conditions [12]. After PAMP receptors recognize their ligands, inflammasomes are activated, and pro-inflammatory caspases cleave GSDMD to break the self-inhibiting structure of the N-terminal and C-terminal regions [30]. Subsequently, GSDMD-NT binds to membrane lipids and creates membrane pores, causing cell lysis and an inflammatory cascade. Pore formation by GSDMD-NT results in the release of IL-1β/-18 and also destroys the homeostasis of water and ions, which ultimately leads to pyroptosis [10]. It has been shown that GSDMD, as a major executor of pyrosis, plays an irreplaceable role in various inflammatory and fibrotic diseases [77]. According to a recent study, GSDMD activation promotes hepatocyte pyroptosis and activates hepatic stellate cells (HSCs), accelerating hepatic fibrosis progression [31]. Mechanistically, HSCs ingest extracellular NLRP3 inflammasome particles, causing GSDMD-pore formation and release IL-1β [31]. Another study indicates that GSDMD knockdown dramatically lowers fibrogenic biomarkers such as procollagen-lysine and TGF-β1, halting knee osteoarthritis and synovial fibrosis [78]. Therefore, conducting research on preventing pyroptosis by inhibiting GSDMD signaling has profound implications for the treatment of fibrosis disease.

GSDME modulates various biological processes, including inflammation and immunology [79]. As previously described, GSDME is cleaved by activated caspase-3 and regulates both pyroptosis and apoptosis [79]. Like GSDMD, the C-terminal domain of this protein inhibits the N-terminal domain. When caspase-3 has been activated, GSDME undergoes cleavage, releasing its N-terminal domain and mediating the formation of pyroptotic pores in cell membranes [79]. Intriguingly, GSDME can also act as an upstream molecule of caspase-3 and regulate apoptotic pathways [80]. In gastric cancer, silencing GSDME can inhibit the development of cancer progression, indicating that GSDME-mediated cancer cell-specific pyroptosis may be a treatment target [81]. Moreover, GSDME also has a significant regulatory function in renal disease. In recent research, GSDME deficiency has been shown to attenuate AKI, and caspase-3/GSDME-induced pyroptosis leads to the occurrence and progression of renal fibrosis [82].

Compared to other gasdermin members, DFNB59 is a non-canonical gasdermin due to the absence of the cleavable linker and the C-terminal autoinhibitory domain [83]. It has been revealed that DFNB59 works as a receptor/adaptor in pexophagy, which maintains the redox balance of auditory hair cells in order to minimize noise-induced damage [83]. Prior to this review, however, it was unclear what role DFNB59 played in pyroptosis and renal fibrosis.

In conclusion, most GSDMs perform an essential function in the process of pyroptosis. Nevertheless, further research is required to determine the specific role of pyroptosis in renal inflammation and fibrosis.

## Gsdmd and caspase-1-dependent classical pyroptotic pathway

**Fig. 4:**
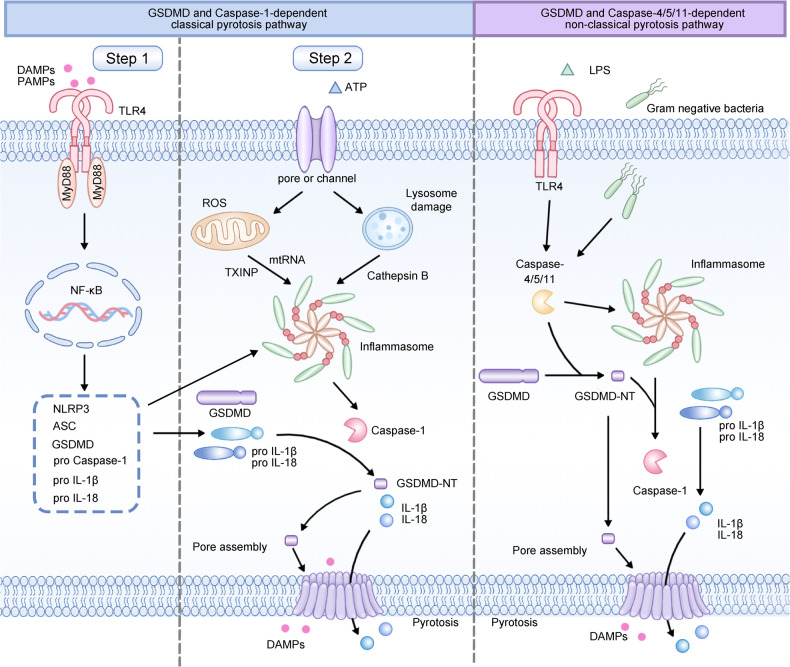
Graphical depiction of the mechanism of classical pyroptosis pathway and non-classical pyroptosis pathway. The classical pyroptosis pathway was activated upon recognition of exogenous and endogenous PAMPs or DAMPs. Two steps mediate the NLRP3 inflammasome activation. In the first step, PAMPs or DAMPs bind to TLRs and activate the MyD88/NF-κB signaling pathway, which results in the production of pro-IL-1β/18. The second step is centered on caspase-1 activation and inflammasome assembly. This step transcriptionally promotes the expression of the NLRP3 gene by activating NF-κB and promotes diverse post-transcriptional alterations of the NLRP3 protein. The NLRP3 oligomerization and ASC recruitment cleavage of the pro-caspase-1 resulted in caspase-1 autocatalytic activation. Importantly, activated caspase-1 cleaves GSDMD to produce GSDMD-NT, which forms plasma membrane pores to induce IL-1β/18 secretions. In more detail, three specific mechanisms are involved in NLRP3 inflammasome assembly. Firstly, extracellular ATP binds to the P2X7 receptor and forms a pore in the cell membrane, resulting in K^+^ efflux and NLRP3 activation. Secondly, particulate or crystalline agonists lead to lysosomal rupture, resulting in the leakage of lysosomal cathepsins B and L that aid in NLRP3 inflammasome assembly. Under the third model, mtDNA and TXNIP stimulate the generation of ROS, which in turn activates the NLRP3 inflammasome. In the non-classical pyroptosis pathway, LPS generated by Gram-negative bacteria activates caspase-4/5 (mouse) or caspase-11 (human) and stimulates the oligomerization and activation of the inflammatory caspases. These active caspases can cleave GSDMD to GSDMD-NT and generate membrane holes, resulting in cascaded responses similar to the classical pyroptosis pathway.

There are two primary pathways that trigger GSDMD-mediated pyroptosis: the caspase-1-dependent classical pyroptotic pathway and the caspase-4/5/11-dependent non-classical pyroptotic pathway [84]. These mechanisms are depicted graphically in Fig. 4.

In the classical pyroptotic pathway, which centers around caspase-1, NLRP3 activation is mediated by the following two steps. In the first step, PAMPs bind to TLRs and activate the MyD88/NF-κB pathway, which leads to the production of pro-IL-1β/18 [85]. This step transcriptionally promotes the expression of the NLRP3 gene by activating NF-κB and promotes diverse post-transcriptional alterations of the NLRP3 protein [86]. The second step is centered on caspase-1 activation and inflammasome assembly [45]. In this step, a combination of PRR, adaptor protein ASC, and pro-caspase-1 aids caspase-1 activation [10]. Importantly, activated caspase-1 cleaves GSDMD to produce GSDMD-NT, which generates 10–14 nm holes in the cell membrane [87]. As a result, GSDMD-NT-mediated pore formation causes IL-1β/18 secretion and disturbs the regulation of water and ions, ultimately leading to pyroptosis [10].

In particular, three main mechanisms have been proposed regarding NLRP3 inflammasome assembly. Firstly, extracellular ATP binds to the ligand-gated ion channel 7 (P2X7) receptor, and the pannexin 1 protein forms a pore in the cell membrane, resulting in potassium efflux and NLRP3 activation [88]. Secondly, crystalline agonists, including silica, amyloid, and uric acid crystals, promote lysosomal rupture, which releases lysosomal cathepsins B and L that aid in NLRP3 inflammasome assembly [89]. Under the third model, mitochondrial DNA (mtDNA) and thioredoxin-interacting protein (TXNIP) promote the production of ROS, which is the common mechanism for assembling the NLRP3 inflammasome [90].

## Gsdmd and caspase-4/5/11-dependent non-classical pyroptotic pathway

In non-classical pyroptotic pathway, caspase-4/5/11 are engaged rather than caspase-1. In this process, TLR4/MD2/CD14 receptors are activated, and LPS is transferred into the cytoplasm by endocytosis and other mechanisms [91]. Consequently, LPS directly activates caspase-4/5/11 by attaching to their CARD domains [34, 92]. In addition, evidence shows that LPS-activated caspase-11 induces K^+^ efflux to activate the NLRP3 inflammasome. After that, active caspase-4/5/11 split GSDMD into GSDMD-NT, resulting in cascaded events analogous to the classical pyroptotic pathway, driving the maturation and release of IL-1β/18 [84, 92].

## Functional role of inflammasomes and pyroptosis in renal inflammation and fibrosis

Although inflammasomes are predominantly expressed in inflammatory cells such as mast cells, lymphocytes, and macrophages, renal resident cells, the most prominent cell type in renal susceptible to renal damage, also contain all parts of the inflammasomes and can secrete mature pro-inflammatory cytokines [47]. Experimental evidence indicates that inflammasome assembly is one of the initial steps in the onset of inflammation and pyroptosis and is essential for renal fibrosis pathogenesis [93]. Upon activation, the inflammasomes promote fibroblasts to convert into myofibroblasts by secreting inflammatory cytokines and pro-fibrotic factors [93].

NLRP3 inflammasome-mediated pyroptosis performs significant regulatory functions in multiple kidney diseases such as unilateral ureteral occlusion (UUO), diabetic nephropathy (DN), obstructive nephropathy, lupus nephritis, and renal fibrosis [90, 93, 94]. Notably, activated NLRP3 inflammasome contributed to the generation of pro-fibrotic factors, and the secretion of IL-1β/18 was an initial and vital occurrence of inflammatory responses in renal fibrosis [95]. In addition, the NLRP3 inflammasome is expressed in kidney tubular epithelial cells and podocytes [96, 97]. Repeated and persistent inflammatory stimuli may promote the development of fibrosis in these cells [96, 97]. In diabetic mice, NLRP3 depletion restored renal function, decreased interstitial fibrosis and inflammation, and suppressed the production of TGF-β1 [98]. A subsequent study showed that HK-2 cells exposed to IL-1β strongly expressed TXNIP, NADPH oxidase 4 (Nox4), and ROS, indicating that inhibition of NLRP3 inflammasome activation suppresses renal fibrosis [97]. Accordingly, Seo et al. demonstrated that NLRP3 inflammasome activation induces UUO-induced renal fibrosis, and gemigliptin reduces pyroptotic activation, which contributes to renal protection [99]. Evidence also shows that the NLRP3 inflammasome regulates lipid metabolism in renal tubular epithelial cells [90]. Mechanically, mitochondrial ROS activates the NLRP3 inflammasome, releasing cytokines that inhibit lipid metabolism and promote pyroptosis [90]. In addition, kidney-specific overexpression of IL-1β was associated with renal inflammation and fibrosis, and IL-1β receptor deficiency protects against mitochondrial dysfunction and NLRP3 inflammasome activation [100]. Clinical trials and real-world studies have revealed that sodium-glucose cotransporter 2 has an advantageous impact on kidney injury, and administration of dagliazine-inhibited NLRP3 inflammasome activation and prevented the development of fibrosis in the kidney [101]. In diabetic mice, lack of carbohydrate response element binding protein decreased renal inflammation and deposition of EMC and restored renal fibrosis [102]. Of note, TGF-β1 stimulated the expression of α-SMA and NLRP3 inflammasome in renal tubule epithelial cells, thus promoting epithelial-to-mesenchymal transition (EMT) and renal fibrosis [18, 102]. Taken together, the aforementioned studies suggest that NLRP3 inflammasome is a crucial contributor among the multiple factors that trigger renal inflammation and fibrosis.

There is also evidence that NLRP1, NLRC4, and AIM2 inflammasomes are associated with certain forms of kidney disease. Soares et al. reported that NLRP1 rs2670660 and rs11651270 polymorphisms were related to a decreased chance of developing DN. This suggested a protective role for NLRP1 in DN and underlined its increasing role as a homeostatic component against metabolic stress [103]. However, the molecular mechanisms underlying its involvement in renal inflammation and fibrosis remain unknown.

The NLRC4 inflammasome is activated by the accumulation of damaged mitochondria, the generation of mROS, and the release of mitochondrial RNA, resulting in the activation of caspase-1 and the secretion of inflammatory cytokines further aggravating kidney injury [56, 104]. Wen et al. revealed that NLRC4 is involved in caspase-1-mediated pyroptosis activation in HK-2 cells exposed to glucose stress [105]. In diabetic rats, treatment with VX-765 suppressed NLRC4 and GSDMD expression, improved renal function, prevented inflammatory cell infiltration, and reduced renal tubulointerstitial fibrosis [105]. Additionally, NLRC4 is one of the hub genes involved in myofibroblasts generation, inflammation, and pyroptosis in renal fibrosis, indicating that NLRC4 is a potential target to inhibit the progression of renal fibrosis [106].

AIM2 was initially classified as a tumor suppressor target; however, it has been discovered that AIM2 also plays a role in renal inflammation and fibrosis [67, 107]. Experimental evidence indicates that DNA fragments from necrotic cells activate the AIM2 inflammasome, ultimately leading to a pro-inflammatory state contributing to renal damage, inflammation, and fibrosis [108]. Moreover, Chung et al. also indicated that AIM2 has a crucial function in glomerular epithelial cell proliferation and modulates ALR-mediated inflammation during glomerulonephritis, indicating that the AIM2-dependent pyroptotic pathway is a potential therapeutic target for proliferative glomerulonephritis and other kidney diseases [109].

In summary, multiple chronic injuries induce inflammasome assembly through the classical and non-classical pyroptotic pathways, contributing to renal inflammation and fibrosis progression.

## Functional role of GSDMS and pyroptosis in renal inflammation and fibrosis

**Fig. 5:**
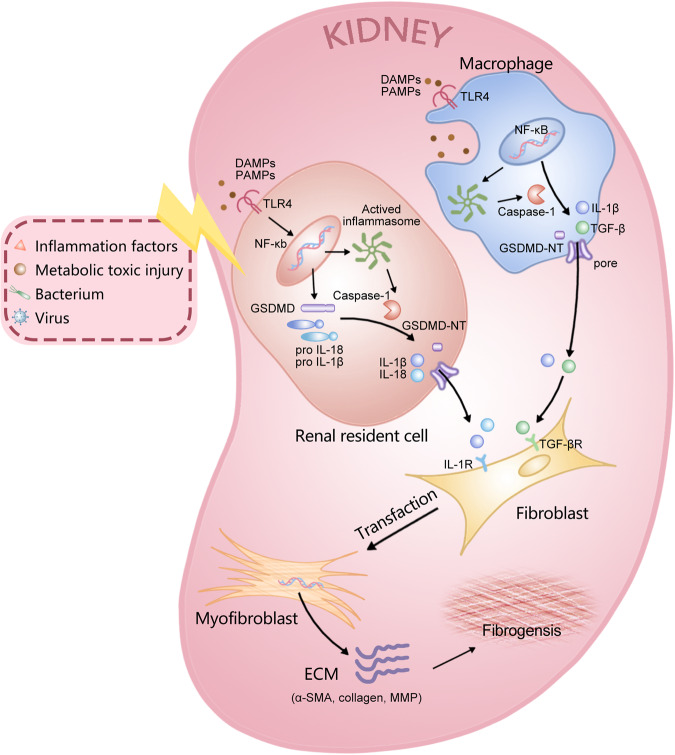
Regulation role of pyroptosis in renal inflammation and fibrosis. Inflammatory factors, bacteria, viruses, toxic metabolic injuries, and other exogenous and endogenous danger signals can induce the initiation of renal inflammation and fibrosis. In this process, renal resident cells and macrophages activate the NF-κB pathway when they recognize DAMPs or PAMPs, which results in the production of pro-IL-1β/18. Subsequently, the activated NLRP3 inflammasome induces pro-caspase-1 autocleavage, and activated caspase-1 cleaves GSDMD to produce GSDMD-NT, which Induces the formation of holes in the plasma membrane. The release of inflammatory cytokines such as IL-1β, IL-18, and TNF-α stimulates the secretion of TGF-1β and increases the expression of the TGF-β receptor. Meanwhile, fibrotic factors such as TGF-1β accelerate the trans-differentiation of fibroblasts into myofibroblasts in the renal stromal region. Myofibroblast activation leads to excessive deposition of ECM, such as α-SMA and collegen, ultimately resulting in renal fibrosis.

Inflammation, infection, immunological response, blood circulation disturbance, and other pathogenic events can stimulate fibroblasts and EMC accumulation in the renal interstitium, resulting in renal fibrosis [110]. GSDMs-dependent pyroptosis has emerged as a crucial inducer of the inflammatory process and takes an essential part in the occurrence and advancement of renal inflammation and fibrosis by secreting a variety of inflammatory cytokines (Fig. 5).

Recent evidence indicates that the UUO activates GSDMD in neutrophils. Of note, GSDMD deletion reduced inflammatory cell infiltration in the kidneys and suppressed extracellular traps, macrophage-to-myofibroblast transition (MMT), and fibrosis formation, revealing the crucial regulatory effect of GSDMD on renal fibrosis [13]. Additionally, as stated previously, VX-765 could alleviate renal damage and fibrosis in diabetes by modulating the GSDMD and caspase-1-dependent classical pyroptotic pathway [105]. Wu et al. reported that GSDMD knockout mice exhibited a significant reduction in albuminuria, azotemia, and kidney fibrosis [111]. Although additional research is required to establish the precise mechanisms mediated by GSDMD in triggering renal inflammation and fibrosis, the current evidence indicates that GSDMD is a crucial component among the multiple factors contributing to this devastating disease.

Unlike GSDMD, GSDME has been demonstrated to be activated by caspase-3 instead of caspase-1/4/5/11. In addition, GSDME-dependent pyroptosis does not require inflammasome assembly, which distinguishes it from GSDMD-dependent pyroptosis [79]. After cleavage by activated caspase-3 and granzyme B, the GSDME-NT domain is released to mediate the formation of pyroptotic pores in cell membranes, resulting in a leak of cellular contents and a subsequent pro-inflammatory response [81]. GSDME is also involved in renal inflammation and fibrosis. In renal tubular epithelial cells, GSDME depletion inhibits cisplatin- or doxorubicin-induced pyroptosis, suggesting that GSDME-targeted treatments may effectively overcome chemotherapeutic drug-associated nephrotoxicity [112]. As early studies reported, Caspase-3/GSDME-dependent pyroptosis initiates ureteral obstruction-induced renal tubule damage, resulting in the development of hydronephrosis, inflammation, and fibrosis in the late stages [112, 113]. In both 5/6 nephrectomy (5/6Nx) and UUO animal models accompanied by CKDs, GSDME knockout improves renal function and prevents the progression of renal fibrosis [114].

In conclusion, GSDMD and GSDME-mediated pyroptosis play critical roles in renal inflammation and fibrosis progression, but exhibit some distinct characteristics (Table 1). However, whether other GSDMs such as GSDMA, GSDMB, GSDMC, and DFNB59 are also associate with the pathologic process of this disease remains unknown.

**Table 1:** Comparison of characteristics between GSDMD and GSDME-mediated pyroptosis involved in renal inflammation and fibrosis.

Pyroptosis mode	GSDMD-mediated pyroptosis	GSDME-mediated pyroptosis	Ref.
Cell types	Renal tubular cell, macrophage, endothelial cell, podocyte	Renal tubular cell, macrophage, podocyte	[13, 16, 77, 82, 112, 113]
Activators	Caspase-1/4/5/11	Caspase-3, granzyme B (GZMB)	[10, 79]
Require inflammasome assemble	Yes	NO	[30, 80]
Cell lysis executor	GSDMD-NT	GSDME-NT	[12, 79]
Pro-inflammation effect	Yes	Yes	[13, 111]
Pro-fibrotic effect	Yes	Yes	[105, 113]
Specific biological function	Promote macrophage-to-myofibroblast transition	Contribute to chemotherapy drug-induced nephrotoxicity	[13, 112]
Potential therapeutic drugs	Disulfiram; NSA	Ac-DMLD-CMK	[30, 112, 140]

## Anti-pyroptotic therapies: emerging pharmacological approaches

As discussed previously in this review, the inflammasomes and pyroptosis are critical in causing renal inflammation and fibrosis, suggesting the possibility of therapeutics targeting the pyroptotic pathway. An in-depth understanding of the mechanisms that regulate pyroptosis will allow for the development of novel targeted inhibitors [115]. Particularly, therapies may target inflammasome assembly, caspase-1 activation, inflammatory cytokines secretion, GSDMD cleavage, and upstream signals (Table 2).

**Table 2:** Potential therapeutic drugs targeting pyroptosis for renal fibrosis.

Therapeutic agents	Targeting molecule	Potential Mechanism	Ref.
MCC950	NLRP3	Binds directly to the NACHT domain of NLRP3, inhibiting ATP hydrolysis and preventing NLRP3 inflammasome formation	[116–118]
Tranilast	NLRP3	Inhibits NLRP3-ASC interactions to prevent NLRP3 assembly	[119–122]
Bay 11-7082	NLRP3	Directly suppress the ATPase activity of NLRP3	[107, 123–125]
Fucoidan	NLRP3	Inhibits AMPK/mTORC1/NLRP3 signaling axis	[93]
Phloretin	NLRP3	Blocks NLRP3 activation and uric acid reabsorption	[126]
Z-YVAD-FMK	Caspase-1	Covalently modifies catalytic cysteine residues in the active site of caspase-1	[128, 129]
VX-765	Caspase-1		[105, 130]
Anakinra	IL-1β	Selective recombinant antagonist of the IL-1β receptor	[131–135]
Canakinumab	IL-1β	IgGκ monoclonal antibody targeting IL-1β	[136, 137]
Disulfiram	GSDMD	Directly target Cys191 of human GSDMD	[30, 138, 139]
NSA	GSDMD	Blocking the oligomerization of p30-GSDMD	[140]
Dihydroquercetin	ROS and NLRP3	Blocking the ROS-associated NLRP3 inflammasome activation	[94, 141]
Complanatoside A	NOX4	Inhibits NLRP3 inflammasome activation and oxidative stress	[142]
AZ10606120	P2X7R	Selective antagonist for P2X7Rs	[144]
A438079	P2X7R	P2X7R antagonist that inhibits potassium (K^+^) efflux	[145]

### NLRP3 inhibitors

Due to the crucial regulatory function of the NLRP3 inflammasome in cell pyroptosis, inhibiting its activation might offer a potential therapeutic approach for renal inflammation and fibrosis. MCC950, a diaryl sulfonylurea-containing compound, is a specific small-molecule inhibitor that blocks NLRP3 inflammasome activation [116]. Mechanistically, MCC950 binds directly to the NACHT domain of NLRP3, inhibiting ATP hydrolysis and preventing NLRP3 inflammasome formation [116]. A recent study found that MCC950 could decrease the expression of NLRP3 inflammasome subunits in renal dendritic cells, thereby preventing renal inflammation and fibrosis [117]. Additionally, intraperitoneal administration of MCC950 to db/db mice reduced serum creatinine and ameliorated renal cortical fibrosis [118]. These observations suggest that the ability of MCC950 to inhibit renal fibrosis is attributable to the inhibition of NLRP3 inflammasome activity. Tranilast is a cell membrane stabilizer that was initially approved to reduce inflammation in allergic conditions [119]. Similar to MCC950, tranilast can prevent the inflammatory activation of NLRP3. Mechanistically, tranilast inhibits NLRP3-NLRP3 and NLRP3-ASC interactions to prevent NLRP3 assembly [120]. Previous research has shown that tranilast prevents pulmonary fibrosis by inhibiting ECM formation via the TGFβ/SMAD2 pathway [121]. As well, tranilast could improve diabetic tubulointerstitial fibrosis in STZ-induced diabetic kidneys and decreases caspase-1 and IL-1β expression [122]. It has been revealed that BAY 11-7082, a sulfonic derivative, could inhibit the nuclear translocation of NF-κB and the NLRP3 inflammasome assembly [123]. Molecularly, Bay 11-7082 inhibits the ATPase activity of NLRP3 and partially inhibits NLRP1 and NLRC4 inflammasomes activation [124]. BAY 11-7082 possesses multiple pharmacological effects, including anti-cancer, neuroprotective, and anti-inflammatory properties [124]. Current evidence revealed that BAY 11-7082 could significantly restore kidney histological architecture and inhibit the serum expression of IL-1β/6 and TNF-α in diabetic rats [107]. In addition, BAY 11-7082 inhibited NLRP3 inflammasome activation and pro-fibrogenic factors expression in the kidney [125]. Wang et al. demonstrated that fucoidan, a class of fucose-rich sulfated carbohydrates, blocks NLRP3 inflammasome-mediated podocyte pyroptosis and fibrosis formation by modulating the AMPK/mTORC1/NLRP3 pathway, being consistent with the effects of MCC950 [93]. Moreover, recent evidence showed that phloretin could effectively attenuate hyperuricemia-induced CKD and renal fibrosis by inhibiting NLRP3 inflammasome activation and uric acid reabsorption. These findings indicated that fucoidan and phloretin may be utilized as NLRP3 inhibitors for the treatment of renal fibrosis [126]. Even though NLRP3 inflammasome inhibitors have promising results in animal models, it remains unclear if these inhibitors can reduce renal inflammation and fibrosis in humans.

### Caspase-1 inhibitors

Caspase-1-mediated pyroptosis promotes the pathological processes of renal fibrosis in animal models, motivating the development of caspase-1 inhibitors as therapeutic medicines [127]. As a selective caspase-1 inhibitor, Z-YVAD-FMK lowered serum levels of caspase-1 and IL-1β/18, improved renal function, and relieved renal damage [128]. In UUO mice model, Z-YVAD-FMK could inhibit caspase-1 expression, reduce ECM accumulation in tubular epithelial cells stimulated by TGF-β1, and suppress the progression of renal tubulointerstitial fibrosis [129]. VX-765 is a potent bioavailable and effective inhibitor of caspase-1. In a mouse model of silicosis, VX-765 can decrease inflammatory lung injury by reducing silica-induced pyroptosis of alveolar macrophages [130]. Mechanically, VX-765 reduces fibrosis by downregulating α-SMA, collagen, and fibronectin. Wen et al. demonstrated that caspase-1 promotes pyroptosis initiation in HK-2 cells, whereas administration of VX-765 to diabetic rats ameliorates renal inflammation and fibrosis through controlling caspase-1-mediated pyroptosis [105]. Therefore, in light of their remarkable efficacy in animal models, future clinical trials with caspase-1 inhibitors are urgently needed to evaluate their efficacy in treating human renal fibrosis.

### IL-1β inhibitors

As noted previously, IL-1β is a significant effector molecule downstream of the pyroptotic pathway and is involved in damage to the kidney, making it a possible therapeutic target for renal fibrosis. Anakinra is a selective IL-1β receptor antagonist for treating inflammatory syndromes such as neonatal-onset multisystem inflammatory illness and rheumatoid arthritis [131]. Anakinra has also been proven to alleviate macrophage infiltration, inflammatory cytokine expression, and ECM accumulation in fibrosis diseases such as liver fibrosis [132], cardiac fibrosis [133], and renal fibrosis [134]. Preclinical studies revealed that Anakinra decreases angiotensin II-induced renal injury in rats, indicating that IL-1β inhibition may protect against kidney injury [135]. Anakinra also decreased blood pressure and renal inflammation in a kidney/DOCA salt-induced hypertension mouse model [134]. However, further and higher-quality clinical trial data are needed to clarify its therapeutic efficacy against renal inflammation and fibrosis. Canakinumab, a monoclonal antibody with a high affinity for IL-1β, proved advantageous for patients with autoinflammatory and cardiovascular diseases. IL-1β inhibition with canakinumab lowers the incidence of severe adverse cardiovascular events in atherosclerotic patients with CKD, particularly in those who had a substantial anti-inflammatory response to early therapy [136]. Recent research indicates that canakinumab is highly effective in treating renal impairment by directly targeting IL-1β [137]. Nevertheless, it remains unknown if canakinumab has a therapeutic effect on renal fibrosis, and further research is eagerly anticipated.

### GSDMD inhibitors

Since the formation of GSDMD-NT pores is a crucial step in pyroptosis, blocking GSDMD by preventing its cleavage or oligomerization is a promising therapeutic strategy. Disulfiram inhibits pyroptosis and permits GSDMD cleavage but prevents IL-1β release and pyroptosis by inhibiting pore formation [138]. Mechanistically, disulfide can directly target Cys191 of human GSDMD [139]. By interfering with the generation of GSDMD pores, disulfiram effectively halts the inflammatory cascade [139]. It has been reported that disulfiram can mitigate the acute renal damage caused by cisplatin in rats by reducing oxidative stress and inflammation [30]. In UUO rats, administration of disulfiram can decrease α-SMA expression and increase E-cadherin level by inhibiting the expression of GSDMD [30]. It was also discovered that necrosulfonamide showed a direct affinity for cleaved GSDMD, blocking the oligomerization of p30-GSDMD and inhibiting the formation of pyroptotic pores [140]. However, additional research is required to evaluate whether this inhibitor is beneficial for renal inflammation and fibrosis.

### Suppression of upstream signals

Due to the crucial biological role of ROS in pyroptosis, inhibition of ROS-associated inflammasome activation is a potential therapeutic strategy. Dihydroquercetin has been demonstrated to have preventive benefits for kidney injuries, including a decrease in urine microalbumin excretion and a reduction in DN-induced renal histopathological damage [94, 141]. One of the potential renal protective mechanisms is the suppression of ROS-associated NLRP3 inflammasome activation [94, 141]. Nicotinamide adenine dinucleotide phosphate (NADPH) oxidase 4 (NOX4) protein activity leads to ROS production and is essential for inducing EMT. Recent evidence revealed that complanatoside A inhibits NOX4-mediated NLRP3 inflammasome activation and oxidative stress, indicating that complanatoside A could be used to treat renal fibrosis [142]. P2X7 receptor (P2X7R) is an upstream molecule of the inflammasome, activated by ATP to promote NLRP3 inflammasome activation [143]. It is currently accepted that P2X7R stimulation can activate various physiological processes, such as immune and inflammatory responses. As an effective inhibitor of P2X7R, AZ10606120 can completely reverse the rise in retinal vascular permeability, IL-6 expression, and VEGF production [144]. A438079, another novel experimental drug, has been shown to block the NF-κB/NLRP3/IL-1β pathway, preventing NLRP3 inflammasome activation and pro-IL-1β/18 expression [145]. Although P2X7R inhibitors have demonstrated promising therapeutic effects in inflammatory disorders, research on their efficacy in animal models of renal inflammation and fibrosis is still insufficient.

## Conclusions and future perspectives

In the past decade, research on the function of pyroptosis in inflammatory disease has advanced substantially. Increasing evidence implicates the activation process of the inflammasome and subsequent pyroptosis as potential regulators of renal fibrosis. As a novel inflammatory form of RCD, pyroptosis plays a double-edged sword role in the progression of renal inflammation and fibrosis. On the one hand, a mild inflammatory response and renal resident cell death are advantageous in protecting the kidneys against pathogen infection, oxidative stress, and other internal and external danger signals. However, excessive and persistent inflammasome activation and pyroptosis of renal resident cells contribute to a severe inflammatory response and accelerate the progression of fibroblast to myofibroblast transformation, eventually leading to renal fibrosis. In this review, we discussed mechanisms involved in the GSDMD/caspase-1-dependent classical pyroptotic pathway and GSDMD/caspase-4/5/11-dependent non-classical pyroptotic pathway, as well as the different regulatory roles of GSDMD and GSDME-mediated pyroptosis in renal inflammation and fibrosis. We also highlighted emerging drugs with the potential to inhibit the pyroptotic pathway for anti-fibrotic therapeutic strategies.

Of note, the NLRP3 inflammasome plays an essential role in renal fibrosis by regulating the activation of caspase-1 and the cleavage of pro-IL-1β/18 in cases of direct injury to the renal. Several inhibitors of the NLRP3 inflammasome, such as MCC950, tranilast, and BAY 11-7082, have been validated for anti-fibrotic effects in vitro and in vivo. However, additional research is required to warrant application in clinical settings. Since GSDMD was confirmed as the specific executor of pyroptosis, GSDMD inhibitors also show potential strategies for alleviating renal inflammation and fibrosis. Although GSDME has also been identified as having a critical function in renal inflammation and fibrosis, the current research on GSDME inhibitors is deficient, so targeting GSDME could serve as a valuable strategy to enhance the development of novel compounds to treat the disease. In addition, other potential strategies, such as microRNA, long noncoding RNAs, antioxidants, and bone marrow-derived mesenchymal stem cells injection, are required for further research.

Overall, it is evident that pyroptosis has yet to disclose all its secrets involved in renal inflammation and fibrosis. Additional research using transgenic animals with pyroptosis defects, such as NLRP3^−/−^ and GSDMD^−/−^ mice, is of great interest to precisely ascertain this question. We believe that pyroptosis-focused research will open up new perspectives for diagnosing and managing renal fibrosis.

## Data Availability

All the data used and analyzed during this study are available from the corresponding author upon reasonable request.
